# Paeoniflorin Effect of Schwann Cell-Derived Exosomes Ameliorates Dorsal Root Ganglion Neurons Apoptosis through IRE1*α* Pathway

**DOI:** 10.1155/2021/6079305

**Published:** 2021-09-27

**Authors:** Yanbo Zhu, Shuo Han, Xiao Li, Yingying Gao, Jiayue Zhu, Xinwei Yang, Liping Xu

**Affiliations:** ^1^School of Traditional Chinese Medicine, Capital Medical University, Beijing, China; ^2^Beijing Key Lab of TCM Collateral Disease Theory Research, Capital Medical University, Beijing, China

## Abstract

**Background:**

Diabetic peripheral neuropathy (DPN) is a common complication of diabetes but its pathogenesis is not fully clarified. Endoplasmic reticulum (ER) stress has been confirmed to be involved in the development of DPN. Dorsal root ganglion neuron (DRGn) is the target cell of DPN injure in the peripheral neurons system. Schwann cell (SCs)-derived exosomes (SC-EXOs) can carry IRE1*α* signal transduction factors in ER stress to DRGn. The aim of this study is to investigate the effect of SC-EXOs treated with paeoniflorin (PF) on DRGn stimulated by high glucose.

**Methods:**

SCs were divided into Control group (Control), 150 mM glucose group (HG), high osmotic pressure group (HOP), and low, middle, and high dose PF group (PF1, PF10, and PF100). Exosomes were obtained from SCs by ultracentrifugation and identified according to marker proteins, including CD63, Alix, Hsp70, and TSG101. ER stress initiating factor GRP78, the IRE1*α* pathway information transmission factor IRE1*α*, and the phosphorylation level of IRE1*α* were detected by Western blot, DRGn is divided into Control group (Control), 50 mM glucose group + Control exosomes group (HG + EXOs Control), 50 mM glucose group (HG), and 50 mM glucose group + administration exosomes group (HG + EXOs PF1, HG + EXOs PF10, and HG + EXOs PF100); ER morphology of primary DRGn was observed by using the transmission electron microscope, the level of DRGn apoptosis was analyzed by TUNEL, and the downstream proteins of ER stress including CHOP, XBP1S, JNK, and p-JNK in DRG and the expression of apoptosis-related proteins Bcl-2, Bax, Caspase-3, and Caspase-12 were measured by Western blot.

**Results:**

Compared with the exosomes in the HG group, the exosomes after the intervention of PF can significantly reduce the expression of GRP78, IRE1*α*, and the phosphorylation level of IRE1*α*(*P* < 0.05); compared with the DRGn in the HG group, the SC-EXOs treated with PF could regulate the expression of proteins downstream of IRE1*α* pathway in ER stress (*P* < 0.05 or *P* < 0.01), improve the morphological integrity of ER, and reduce apoptosis in DRGn (*P* < 0.05 or *P* < 0.01).

**Conclusion:**

PF regulates the information of ER stress carried by SC-EXOs and further affects downstream of IRE1*α* pathway in DRGn, thus reducing ER stress-induced apoptosis. PF can interfere with DPN through affecting information communication carried by EXOs between SCs and DRGn.

## 1. Introduction

Diabetic peripheral neuropathy (DPN) is one of the most common long-term complications of diabetes, with a prevalence of over 50%, and is characterized by sensory and motor neuron damage [1, 2]. The pathogenesis of DPN is complex, including microvascular ischemia, polyol pathway, oxidative stress, and endoplasmic reticulum stress (ER stress) [3]. Our previous studies have shown that apoptosis induced by ER stress is one of the important mechanisms of the pathogenesis of DPN [4–6]. Endoplasmic reticulum (ER) is an important organelle for protein synthesis, folding and modification, lipid synthesis, and calcium storage. ER stress is caused by the accumulation of unfolded or misfolded proteins, interruption of lipid synthesis, or depletion of calcium storage. High glucose stimulation can cause ER stress in cells, and the cells respond to ER stress through unfolded protein response (UPR) to maintain ER homeostasis. UPR mainly contains PERK, ATF6, and IRE1*α* signal pathways [7]. IRE1*α* pathway is the most conservative branch of UPR in evolution, which can reduce the protein folding load and increase the ER proteins folding ability [8].

Dorsal root ganglion (DRG) is one kind of cell bodies, and the susceptibility of dorsal root ganglion neurons (DRGn) to high glucose concentration stress in vivo and in vitro is involved in the occurrence and development of diabetic neuropathy. At the same time, ER stress can also cause abnormal DRGn ion channel function, gene expression, transcription regulation, metabolism, and protein folding [9].

As a kind of lipid bilayer membrane vesicles involved in cell-to-cell communication, exosomes play a key role in intracellular communication [10]. Exosomes are wrapped in bilayer membranes to protect their genetic material. In this way, exosomes can be used as stable and effective carriers to carry specific goods such as proteins, lipids, and genetic materials, so they can be used as a promising tool to transport anti-infective goods to target tissues or organs [11]. Extracellular pathway is an effective way to regulate apoptosis, angiogenesis, and target cell inflammation [12]. The SC-EXOs can significantly promote axonal growth and regeneration both in vitro and in vivo [13, 14]. Axon regeneration is an important process for functional recovery after being stimulated by high glucose [15, 16]. Therefore, SC-EXOs may be used as carriers to participate in the regulation of DRGn axon regeneration and growth and promote the repair of peripheral nerves after injury [17]. The study showed that the secretion of exosomes would increase under ER stress, but in the IRE1*α*-deficient cells, the secretion of exosomes did not increase. This indicated that the IRE1*α* pathway was involved in the release of exosomes [18]. At the same time, with ER stress, the endoribonuclease activity activated by IRE1*α* will cleave XBP1 (X-box binding protein 1) mRNA which could play the role of transcription factor. The IRE1*α*-XBP1 pathway was involved in insulin resistance and dyslipidemia. It has been previously demonstrated that IRE1*α* was essential for the release of exosomes from palmitate-treated liver cells [19, 20]. However, the role of exosomes stimulated by IRE1a and the regulation of the IRE1a pathway are still unclear.

Paeoniflorin (PF) is a monoterpene glycoside isolated from Ranunculaceae plant *Paeonia lactiflora*, which acts as antioxidant and anti-inflammatory and performs vasodilatation and other biological activities [21]. PF can block the PERK pathway by inhibiting the expression of GRP78 and finally reduce ER stress [22]. At the same time, the anti-inflammatory effect of PF is achieved by inhibiting the phosphorylation level of IRE1*α* [23, 24]. Therefore, this article studies the effect of PF on SC-EXOs in IRE1*α* signaling pathway of DRGn.

## 2. Materials and Methods

### 2.1. Primary DRGn Extraction and SCs Culture

DRGn was prepared from SD neonatal rats regardless of gender (3–5 days old, Charles River Experimental Animal Technology Co., Ltd.). The DRG is taken from the intervertebral foramen of both vertebrae. The tail of the DRG is cut off, and its head is retained. The head is cut into pieces and incubated in 4 mL trypsin with 1% collagenase for 1-2 hours at 37°C. Dissociated cells were seeded in Petri dish coated with poly-L-lysine (PLL, Sigma-Aldrich), incubated for 7 days, and the medium is replaced every 2-3 days [25, 26].

SCs line was cultured in DMEM complete medium containing 10% fetal bovine serum (FBS) and 1% penicillin-streptomycin. The incubator was kept at 37°C and 5% CO_2_. They were divided into Control group (25 mmol/L glucose DMEM ＋ 10% FBS, Control), high glucose group (150 mmol/L glucose DMEM ＋ 10% FBS, HG), high osmotic pressure group (25 mmol/L glucose DMEM ＋ 44.4 mmol/L Mannitol ＋ 10% FBS, HOP), PF low dose group (150 mmol/L glucose DMEM ＋ 10%FBS + 1 *μ*M PF, PF1), PF medium dose group (150 mmol/L glucose DMEM ＋ 10%FBS+10 *μ*M PF, PF10), and PF high dose group (150 mmol/L glucose DMEM ＋ 10%FBS + 100 *μ*M PF, PF100). After 24 hours of modeling, the supernatant was collected to extract exosomes.

### 2.2. Exosomes Extraction

SC-EXOs were extracted by ultracentrifugation from the culture supernatants. SC-EXOs were extracted by ultracentrifugation from the culture supernatants. The supernatants were collected and sequentially centrifuged firstly at 300 × g for 10 min, then the supernatants were collected and secondly at 2,000 × g for 20 min. Then again they were collected; next 30 min with 10,000 × g. The supernatant was transferred into the 8.9 mL centrifugal tube (Beckman, USA) to ultracentrifugation at 100,000 × g for 70 min twice at 4°C to get the exosomes. Our extraction method was the same as described in the previous study [27]. After sucking out all the supernatant, the precipitate was dissolved in 200 *μ*L PBS and collected in 1.5 mL centrifuge tube for follow-up experiments.

### 2.3. Cell CCK8 Assay

100 uL SCs suspension was inoculated in each well of 96-well plate, and Control group (25 mmol/L glucose DMEM ＋ 10% FBS), HG group (150 mmol/L glucose DMEM ＋ 10% FBS, HG), and HOP group (25 mmol/L glucose DMEM ＋ 44.4 mmol/L Mannitol ＋ 10% FBS) were set up. Six multiple holes were set in each group. After 24 hours, 10 *μ*l CCK8 solution was added to each hole. The culture plate was incubated in the incubator for 3 hours. The absorbance value was measured by enzyme labeling instrument at 450 nm. Cytoactive rate^*∗*^(%) = [(As − Ab)/(Ac − Ab)] × 100%, where As is HG or HOP group absorbance; Ac is Control group absorbance; and Ab is Blank hole absorbance.

### 2.4. Confocal Laser Microscopy

The primary DRGn was identified, and the SC-EXOs internalized by DRGn were observed with immunofluorescence.

DRGn identification: DRG was extracted and inoculated into cell climbing tablets and cultured for 7 days. DRGn was stained with NF-H polyclonal antibody (18934-1-AP, Proteintech, NF200) for specific immunofluorescence staining, and the morphology of DRGn was observed by using laser confocal microscope.

DRGn internalized SC-EXOs: DRGn was stained by immunofluorescence with NF-H polyclonal. The exosomes were labeled with PKH26 (MINI26-1KT, Sigma) and co-cultured with DRGn for 24 hours. The situation of SC-EXOs internalized by DRGn was observed by immunofluorescence.

### 2.5. Transmission Electron Microscopy

The primary DRGn was inoculated on a six-well culture plate and cultured for 7 days. DRGn collected by centrifugation was fixed with glutaraldehyde for 2 hours, and the samples were rinsed 3 times with phosphate buffer. It is taken to Core Facility Center of Capital Medical University. The ER in DRGn was observed by using the HT7700 transmission electron microscope (Hitachi, Japan). The clear ER image was finally obtained by using the CCD camera and the microscope host.

### 2.6. Western Blot

The treated DRGn and SC-EXOs were collected. Cellular protein was extracted by RIPA lysis buffer. The equal proteins were separated on an SDS-PAGE, and then they are transferred to the PVDF membrane. The membrane was blocked with 5% nonfat dry milk for 1 hour. The PVDF membrane was incubated overnight at 4°C with primary antibodies, including Alix (1 : 2000, ab275377, Abcam), Hsp70 (1 : 2000, ab2787, Abcam), CD63 (1 : 2000, ab134045, Abcam), TSG101 (1 : 2000, ab125011, Abcam), GRP78 (1 : 2000, ab108613, Abcam), IRE1*α* (1 : 500, sc-390960, Santa cruz), p-IRE1*α* (1 : 2000, ab124945, Abcam), CHOP (1 : 500, sc-46661, Santa cruz), JNK (1 : 2000, ab208035, Abcam), p-JNK (1 : 2000, ab124956, Abcam), XBP1 (1 : 500, sc-8015, Santa cruz), Bcl-2 (1 : 2000, ab182858, Abcam), Bax (1 : 2000, ab32503, Abcam), Caspase-3 (1 : 2000, ab32351, Abcam), Caspase-12 (1 : 2000, ab62484, Abcam), and *β*-actin (1 : 2000, ab8226, Abcam). The membranes were incubated with the appropriate horseradish peroxidase-conjugated secondary antibodies. The specific bands were observed by using the hypersensitive ECL chemiluminescence kit (New Cell & Molecular Biotech Co., Ltd, P10200).

### 2.7. TUNEL Assay

DRGn was cultured in groups on the cell climbing slice; the cells were fixed by 4% paraformaldehyde, 0.1%TritonX-100 permeated through the membrane, dripped with TUNEL reaction solution (G1501-50T, Servicebio), and incubated at 37°C for 2 h. The nuclei were stained with DAPI and then sealed with antifluorescence quenchant. Under the 880 Airyscan laser confocal microscope (Zeiss, Germany), 8 visual fields were randomly selected in each group, the number of apoptotic cells and total cells were counted, and the percentage of apoptosis was calculated.

### 2.8. Statistical Analysis

The software used to process data includes GraphPad Prism8.0 and Image J. The results are shown as mean ± standard error of mean (SEM). Differences were analyzed by one-way ANOVA followed by Tukey's multiple comparisons test. Student's unpaired *t*-test was used to analyze data between 2 groups. *P* < 0.05 was considered statistically significant.

## 3. Result

### 3.1. The Cytoactivity of SCs Was Not Affected by High Osmotic Pressure

**Figure 1:**
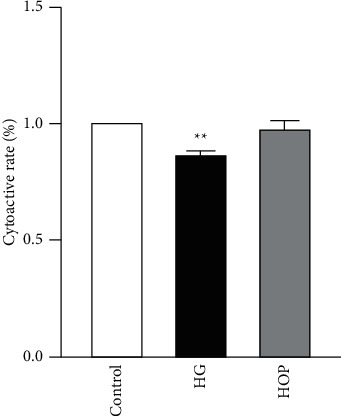
Determination of SCs high glucose and high osmotic pressure cell cytoactivity by CCK8 ^*★★*^*P* < 0.01 vs. Control, *n* = 6 for each group.

Compared with Control group, the cytoactivity in HG group was significantly decreased (*P* < 0.01), while the HOP group had no significant difference (Figure 1). This experiment confirmed that high osmotic pressure had no effect on cell cytoactivity. The changes in SCs and SC-EXOs were caused by high glucoses concentration, not osmotic pressure.

### 3.2. Identification of SC-EXOs Incubated in High Glucose

**Figure 2:**
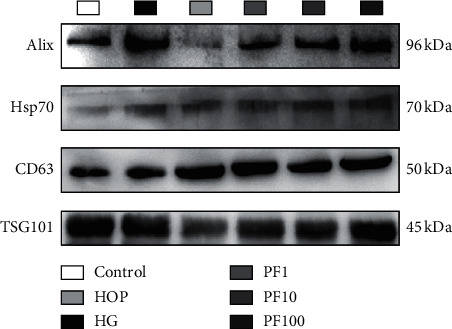
Exosomes marker protein of SC-EXOs. Western blot showed specific markers Alix, CD9, Hsp70, CD63, and TSG101 in exosomes.

Figure 2 shows the results of Western blot. The exosomes specific proteins Alix, Hsp70, CD63, and TSG101 were detected. They were expressed in the exosomes of each group. The expression of the marker protein indicated the presence of exosomal components which proved that SCs can secrete exosomes, and using the ultracentrifugation to extract exosomes was reliable.

### 3.3. Identification of Primary DRGn and Internalization of SC-EXOs

**Figure 3:**
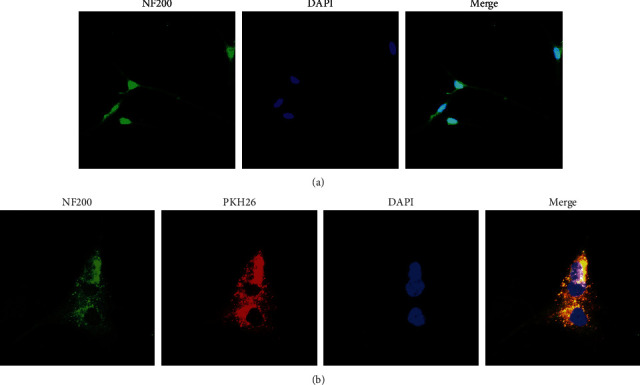
Identification of the DRGn and the internalization of SC-EXOs. (a) NF200 specific identification of DRGn (× 200), scale bar, 100 *μ*m. (b) DRGn internalized SC-EXOs (× 400), scale bar, 50 *μ*m.

Identification of primary DRGn: in order to verify whether the primary DRGn could be grown adherently after extracting the DRG by our method, we used the currently recognized DRGn specific antibody NF200 for identification. Cultured for 7 days, the immunofluorescence results showed that the adherent cells could appear green fluorescence, indicating that the adherent cells could bind to the specific antibody NF200, and the cells also showed the characteristics of DRGn: the whole cell was triangular and had slender axons (Figure 3(a)). This proved that our extraction method is correct, and the cell was primary DRGn.

The DRGn showed green fluorescence, and the nucleus showed blue fluorescence by using the laser confocal microscope. The exosomes showed red fluorescence. After merging, the red exosomes were located in the cytoplasm of green DRGn cells and distributed around the blue DRGn nucleus (Figure 3(b)). This proved that DRGn can internalize exosomes.

### 3.4. PF Regulated GRP78 and IRE1*α* in SC-EXOs Incubated in High Glucose

**Figure 4:**
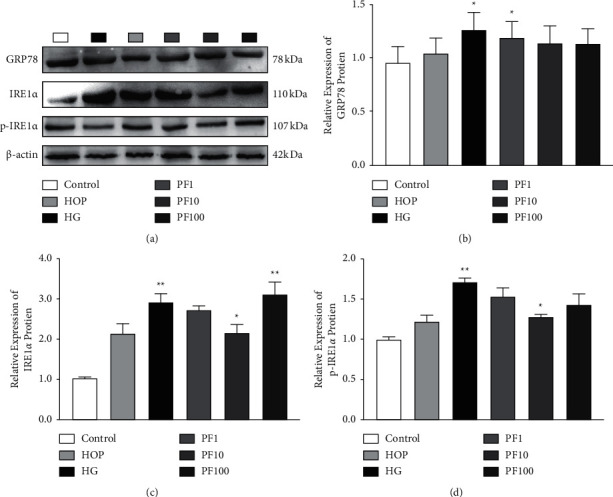
PF interfered with SC-EXOs carrying ER stress information. (a) The expression of ER stress initiating factors in exosomes. The protein expression level for (b) GRP78, (c) IRE1*α,* and (d) p-IRE1*α* was determined by Western blotting, *n* = 4 for each group. The results are expressed as the mean ± standard error.

Compared with the Control group, the expression of GRP78 in the HG group increased (*P* < 0.05); compared with the HG group, the expression of the PF1 group decreased (*P* < 0.05; Figure 4(b)). Compared with the Control group, the expression of IRE1*α* and p-IRE1*α* in the HG group increased (*P* < 0.01); compared with the HG group, the PF10 group decreased (*P* < 0.05; Figures 4(c) and 4(d)). Thus, it could be seen that high glucose can lead to ER stress, PF can play a role in anti-ER stress, and exosomes participate in the information transmission of IRE1*α* pathway.

### 3.5. Effect of PF Intervention of SC-EXOs on the Downstream Protein Expression of IRE1*α* Signal Pathway in DRGn

**Figure 5:**
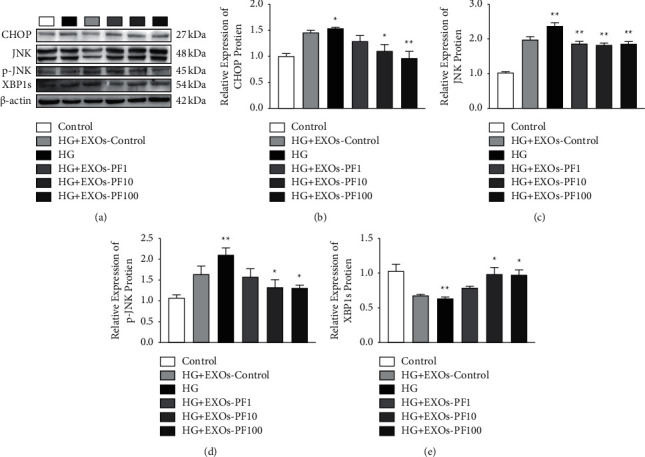
The effect of PF interfered with SC-EXOs on the downstream proteins of ER stress in DRGn. (a) The expression of ER stress downstream protein in DRGn. The expression level of (b) CHOP, (c) JNK, (d) p-JNK, and (e) XBP1s was determined by Western blotting, *n* = 4 for each group. The results are expressed as the mean ± standard error.

CHOP, XBP1s, C-Jun N-terminal kinase (JNK), and p-JNK are regarded as the downstream indexes of ER stress IRE1*α* pathway [28, 29]. We verify their expression by Western blot. Compared with the Control group, the expression of CHOP, JNK, and p-JNK increased, and the expression of XBP1s decreased in the HG group (*P* < 0.05 or *P* < 0.01). Compared with the HG group, the expression of CHOP, JNK, and p-JNK significantly decreased and the expression of XBP1s increased in the exosomes intervention group (*P* < 0.05 or *P* < 0.01; Figures 5(b)–5(e)). This proved that the exosomes after the intervention of PF can reduce the ER stress.

### 3.6. Effect of PF Intervention of SC-EXOs on the Morphological Integrity of ER in DRGn

**Figure 6:**
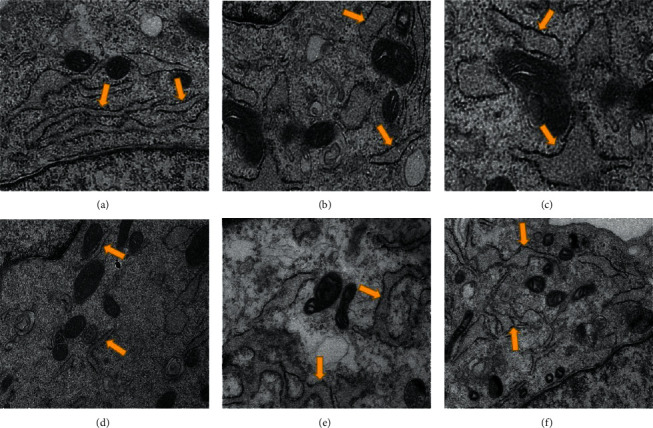
Ultrastructural observation of ER (7,000x). The yellow arrow refers to the ER. (a–f) The Control group, HG + EXOs Control group, HG group, HG + EXOs PF1 group, HG + EXOs PF10 group, and HG + EXOs PF100 group.

We can see that the shape of ER in the Control group is uniform (Figure 6(a)). After 24 hours of 50 mM glucose intervention, the morphology of ER was partially enlarged, broken, and fragmented (Figure 6(c)). In the 50 mM glucose + EXOs PF group, the structure of ER tended to be intact (Figures 6(d) and 6(e)). This can explain that the PF interferes with SC-EXOs to maintain the intact morphology of ER. The morphology of ER in SC-EXOs PF100 and SC-EXOs Control group has improved but the effect was not obvious (Figures 6(b) and 6(e)).

### 3.7. PF Interfered with SC-EXOs to Reduce Apoptosis of DRGn

#### 3.7.1. PF Interfered with SC-EXOs on Apoptosis of DRGn in High Glucose Concentration by TUNEL Kit

**Figure 7:**
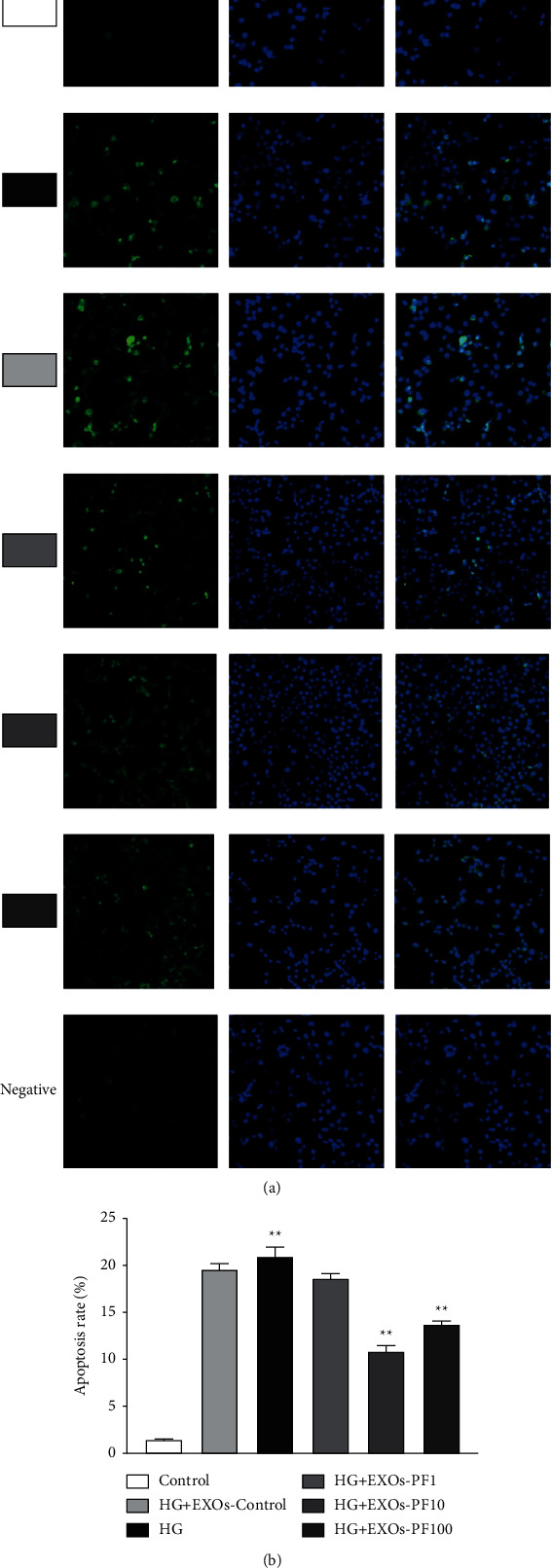
The apoptosis level of DRGn was detected by immunofluorescence (× 200) dcale bar, 100 *μ*m. The green fluorescence was the apoptotic cells stained by TUNEL kit, and the blue fluorescence was the nucleus. Blue-green overlap was the apoptotic cells. *n* = 6 for each group.

The TUNEL kit method is currently recognized as one of the methods to detect the level of apoptosis. Compared with the Control group, a large number of green fluorescence appeared in the HG group (Figure 7(a)), and the level of apoptosis increased in the HG group (*P* < 0.01; Figure 7(b)). Compared with the HG group, different doses of PF interfere with the SC-EXOs to reduce apoptosis of DRGn (*P* < 0.01; Figure 7(b)). Compared with the HG + EXOs PF1 group, the apoptosis rate of DRGn in the HG + EXOs PF10 group and HG + EXOs PF100 group was significantly decreased (*P* < 0.01). The intervention effect of PF with doses of 10 *μ*M and 100 *μ*M was the best.

#### 3.7.2. PF Interfered with SC-EXOs on DRGn Apoptotic Protein in High Glucose Concentration

**Figure 8:**
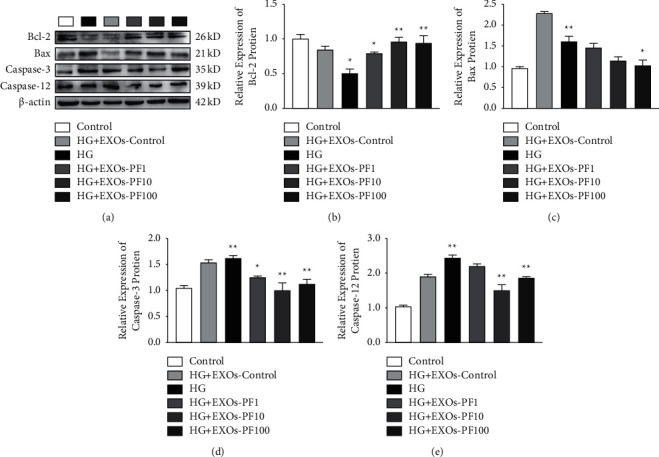
The effect of PF interfered with SC-EXOs on the apoptotic proteins in DRGn. (a) The expression of apoptotic protein in DRGn. DRGn apoptosis protein expression for (b) Bax, (c) Bcl-2, (d) Caspase-3, and (e) Caspase-12 was determined by the western blot. The results were expressed as the mean ± standard error, *n* = 4 for each group.

In addition to using the TUNEL kit to detect the degree of apoptosis in DRGn, we also verified the expression of anti-apoptotic protein Bcl-2 and pro-apoptotic protein Bax and Caspase family proteins Caspase-12 and Caspase-3 by Western blot. Compared with the Control group, the expression of Bax, Caspase-3, and Caspase-12 was significantly increased and the expression of Bcl-2 was decreased in the HG group (*P* < 0.05 or *P* < 0.01); compared with the HG group, the expression of Bax, Caspase-3, and Caspase-12 was significantly decreased and the expression of Bcl-2 was significantly increased in the HG + EXOs PF group (*P* < 0.05 or *P* < 0.01; Figures 8(b)–8(e)). Western blot showed that EXOs interfered with PF could inhibit the expression of apoptotic protein in DRGn, which was consistent with the results of the previous TUNEL kit. It was more confirmed that EXOs after the intervention of PF can reduce the apoptosis induced by high glucose.

## 4. Discussion

In diabetic peripheral neuropathy, the research group has found that exosomes could play the role of antioxidative stress through information communication and then improve DPN. There was a variety of pathogenesis of DPN. The studies have shown that exosomes can improve DPN by regulating the communication between SCs and DRGn. ER stress is one of the pathogenesis of DPN [30]. PF can improve the apoptosis of SCs in high glucose concentration by reducing ER stress and has the potential to interfere with DPN [6]. However, can PF interfere with DPN by improving the communication between SCs and DRGn? What is the specific way of action? These have not been fully clarified at present. Our study confirmed that PF can reduce the apoptosis of DRGn by regulating the signal proteins related to ER stress in SC-EXOs.

In this study, ER stress was used as a breakthrough point to further explain the role of SC-EXOs. We used an in vitro model to verify this hypothesis. The results of this study are as follows: (1) SC-EXOs can carry ER stress initiating factor and IRE1*α* pathway initiating factor; (2) the hyperosmotic condition does not affect the activity of SCs and the SC-EXOs; (3) DRGn can internalize the SC-EXOs and use the exosomes communication mechanism to transmit information; (4) high glucose can aggravate the ER stress and change the morphology of ER in DRGn; and (5) SC-EXOs under the intervention of PF can reduce the apoptosis of DRGn in high concentration.

DPN as a disease of the peripheral nervous system, high glucose stimulation can cause damage to SCs and DRGn axons [31]. SC-EXOs can promote the activity of nerve cells [32]. At the same time, the SC-EXOs also can significantly promote axonal growth and axonal regeneration. Axon regeneration is an important process of functional recovery after peripheral nervous system injury [33]. Therefore, the SC-EXOs may be involved in the axonal regeneration and growth regulation of DRGn and promote the repair of peripheral nerve injury. Then, the molecular mechanism of SC-EXOs is explained to promote DRGn axonal growth, and thus ER stress is inhibited to reduce cell apoptosis; this is the core problem solved in this study.

We previously found that hyperglycemia can cause axonal damage in DRGn and trigger DPN. The degeneration of distal sensory axons in DRGn is the main cause of DPN [34, 35]. SCs can interact with the axons of DRGn, and SCs in the normal concentration can secrete exosomes to promote the proliferation of injured DRGn [36]. SC-EXOs were internalized by DRGn axons, and miR-27a in exosomes promoted neurite growth of diabetic DRGn [30]. Our results showed that DRGn could internalize SC-EXOs, and exosomes were involved in the recovery of damaged DRGn. However, our results showed that the exosomes under the intervention of PF significantly alleviated the ER stress and apoptosis of DRGn. SC-EXOs under normal conditions can have a therapeutic effect on DRGn stimulated by high glucose, but the results are not significantly different. Therefore, it can be shown that PF intervention can help exosomes carry IRE1*α* pathway information to inhibit apoptosis.

It has been proved that the condition of high glucose concentration can increase the number of exosomes and interfere with the biological activity of exosomes [37]. SCs with axonal damage can also release a large number of exosomes [38]. Therefore, it is proved that high glucose concentration can affect the exosomes, and we have previously proved that high glucose concentration can increase the expression of exosomal marker protein CD63 compared with the Control group [39], which is consistent with our experimental results. In order to eliminate the effect of other conditions on the exosomes derived from SCs, we also treated SCs with high osmotic pressure. At the same time, we tested the SCs and SC-EXOs after intervention and found that high osmotic pressure did not affect the activity of SCs and the information transmission between exosomes and DRGn.

The research group previously proved that DPN was characterized by demyelination caused by SCs apoptosis induced by ER stress. The pathogenesis of DPN is complicated, but ER stress is closely related to apoptosis. In the process of UPR, IRE1*α* dissociates with GRP78 and then undergoes autophosphorylation to activate downstream reactions [40]. The results showed that both IRE1*α* and GRP78 were expressed in exosomes, and their expression increased significantly in high glucose concentration, indicating that exosomes can carry information transmission factor and activate IRE1*α* signal pathway. At the same time, the expression of both exosomes decreased after the intervention of PF. C/EBP homologous protein (CHOP), as a downstream protein of ER stress, is upregulated in SCs of the diabetic nephropathy rat model. Under ER stress, activated IRE1*α* can no longer regulate the expression of transcription factors CHOP and Caspase-12 and further activate the expression of Caspase-3, aggravating apoptosis-mediated DPN damage. All these are consistent with our experimental results. CHOP played a key role in cell apoptosis induced by ER stress. It was considered to play a central role in cell apoptosis induced by ER stress. It is strongly induced by IRE1*α* signal. Inhibiting the expression of IRE1*α* can inhibit the expression of CHOP [41]. At the same time, in the diabetic model, the expression level of XBP1s in hippocampus decreased significantly, suggesting that the decrease in XBP-1s expression is closely related to the damage of the peripheral nervous system caused by diabetes [42]. Excessive or persistent ER stress can also activate the phosphokinase activity of IRE1*α*. Activated ASK1 induces and increases the expression of JNK by increasing the transcription of JNK. It has been found that the expression of JNK is significantly decreased after the application of IRE1*α* phosphokinase inhibitor [43]. Our results of Western blot also show that PF interferes with SC-EXOs to affect the expression of ER stress downstream factors in DRGn, inhibits the expression of apoptotic factors Caspase-12 and Caspase-3, reduce apoptosis, and alleviates ER stress by exosomes.

In summary, our study shows that SCs can release exosomes, and the method of extracting exosomes by ultracentrifugation is feasible. Exosomes can carry ER stress initiating factor and IRE1*α* signaling pathway information transmission factor for cell-to-cell information exchange. High glucose concentration will affect SCs secretion of exosomes, while high osmotic pressure will not affect SCs activity and exosomes secretion. PF can improve the ER stress of DRGn and affect the expression of downstream proteins such as JNK and CHOP; improve the morphology in ER of DRGn damaged by high glucose; and increase the expression of anti-apoptotic protein Bcl-2, decrease the expression of pro-apoptotic protein Bax, inhibit the expression of Caspase family Caspase-12 and Caspase-3, and ultimately improve DPN. As a carrier of cellular communication, exosomes provide new ideas for the treatment of DPN, diabetes, and its complications.

## 5. Conclusion

This study proved that SCs can secrete exosomes. PF can affect the expression of IRE1*α* and GRP78 which were the key proteins of IRE1*α* signal pathway in exosomal ER stress. DRGn can absorb SC-EXOs. PF carries ER stress information by exosomes, which plays the role of anti-ER stress and reduces DRGn apoptosis, thus ameliorating DPN. Our research provided a new method for the treatment of DPN from the molecular mechanism level.

## Data Availability

The data used to support the results of this study are included in the article.
